# Defective Autophagosome Formation in p53-Null Colorectal Cancer Reinforces Crocin-Induced Apoptosis

**DOI:** 10.3390/ijms16011544

**Published:** 2015-01-09

**Authors:** Amr Amin, Khuloud Bajbouj, Adrian Koch, Muktheshwar Gandesiri, Regine Schneider-Stock

**Affiliations:** 1 Department of Biology, College of Science, United Arab Emirates University, Al-Ain 15551, United Arab Emirates; E-Mail: khuloud@hotmail.de; 2 Zoology Department, Faculty of Science, Cairo University, Cairo 12613, Egypt; 3 University of Hawaii Cancer Center, University of Hawaii, Honolulu, HI 96822, USA; 4 Experimental Tumor Pathology, Institute of Pathology, University of Erlangen, Erlangen 91054, Germany; E-Mails: adrian.koch@uk-erlangen.de (A.K.); mukthi_14@yahoo.com (M.G.)

**Keywords:** autophagy, autophagosome, apoptosis, crocin, colorectal cancer, p53

## Abstract

Crocin, a bioactive molecule of saffron, inhibited proliferation of both HCT116 wild-type and HCT116 p53−/− cell lines at a concentration of 10 mM. Flow cytometric analysis of cell cycle distribution revealed that there was an accumulation of HCT116 wild-type cells in G_1_ (55.9%, 56.1%) compared to the control (30.4%) after 24 and 48 h of crocin treatment, respectively. However, crocin induced only mild G2 arrest in HCT116 p53−/− after 24 h. Crocin induced inefficient autophagy in HCT116 p53−/− cells, where crocin induced the formation of LC3-II, which was combined with a decrease in the protein levels of Beclin 1 and Atg7 and no clear p62 degradation. Autophagosome formation was not detected in HCT116 p53−/− after crocin treatment predicting a nonfunctional autophagosome formation. There was a significant increase of p62 after treating the cells with Bafilomycin A1 (Baf) and crocin compared to crocin exposure alone. Annexin V staining showed that Baf-pretreatment enhanced the induction of apoptosis in HCT116 wild-type cells. Baf-exposed HCT116 p53−/− cells did not, however, show any enhancement of apoptosis induction despite an increase in the DNA damage-sensor accumulation, γH2AX indicating that crocin induced an autophagy-independent classical programmed cell death.

## 1. Introduction

Colorectal cancer (CRC) has the fourth most common malignant tumors worldwide [1]. Although CRC mortality has been progressively declining since 1990, it still remains the second most common cause of cancer death in the US and Europe [2]. Approximately 150,000 Americans and 250,000 Europeans are diagnosed with CRC annually, and about one-third of those die of the disease [3]. Surgery is the primary treatment for colorectal cancer. Chemotherapy is used to reduce the likelihood of metastasis development, shrink tumor size, or slow tumor growth. Adjuvant chemotherapy has been recommended for stage III colon cancer patients [4]. However, the overall survival advantage in CRC patients treated with neoadjuvant therapy remains disappointing. Therefore, in the new era of personalized cancer medicine, chemotherapy continues to be essential for treating patients with CRC and advent of novel cytotoxic biomolecules is desperately needed. Thus, the search for chemotherapeutic agents for the treatment of colorectal cancer is highly warranted [5]. Such a potential chemotherapeutic agent is the saffron-based crocin. Saffron (*Crocus sativus* L.) is traditionally used as a coloring or flavoring agent and its constituents, including crocin, crocetin, picrocrocin, and safranal, have all demonstrated health promoting properties. Although many studies support the notion that saffron may be a promising cancer therapy agent, its detailed molecular mechanisms are still lacking. Saffron was also proposed as a good apoptotic inducer of tumor cells. Saffron’s ability to induce apoptosis has been reported to play a crucial role in the death of human cervical carcinoma cells (HeLa), human hepatocellular carcinoma cells (HepG2), and human colorectal cancer cells [6,7,8]. *In vitro* studies have shown that saffron’s major carotenoid, crocin, is regarded as the most promising anticancer compound in saffron as it has been reported to have inhibitory effects against a wide range of cancer cells including human cervical carcinoma HeLa cells, adenocarcinoma cells, and different types of breast cancer cells [9,10].

Several groups have reported that most of the cellular systems in which autophagy was proven to contribute to cell death had defects in the apoptosis signaling pathway [5,11,12]. To that end, the tumor suppressor protein p53 is known to induce and/or repress the expression of target genes involved in central pathways, such as control of the cell cycle and apoptosis [13]. p53 triggers cell cycle arrest and hence allows DNA damage repair or promotes apoptosis if cells are challenged with severe irreparable insults [14]. Mutations in the p53 gene are detected in most CRCs and are thought to be late events in the transition from dysplastic adenomas to invasive carcinomas [15,16]. The connection between apoptotic and autophagic cell death in the context of cancer is still unresolved. Autophagy provides cancer cells with a protective response under unfavorable conditions [17]. During cellular stress, cells utilize autophagy to adapt to the microenvironment, but autophagy due to excessive stress leads to cell death [18,19,20]. On the other hand, several studies have reported that autophagy is triggered in some cancers in response to various anticancer agents, including As_2_O_3_, tamoxifen, and temozolomide [21,22,23]. The cellular function of autophagy remains a matter of debate, and its role in cancers is still particularly controversial [20].

This study is set to introduce crocin as a potential chemotherapeutic agent for colorectal cancer where the molecular mechanism through which crocin induces cell death in two p53 isogenic HCT116 cells is investigated. Our results unveil a novel mechanism of action of crocin in inducing autophagy and/or apoptosis in human colon cancer cells in a p53-dependent manner.

## 2. Results

### 2.1. Crocin Inhibits Proliferation of HCT116 Cell Lines

**Figure 1:**
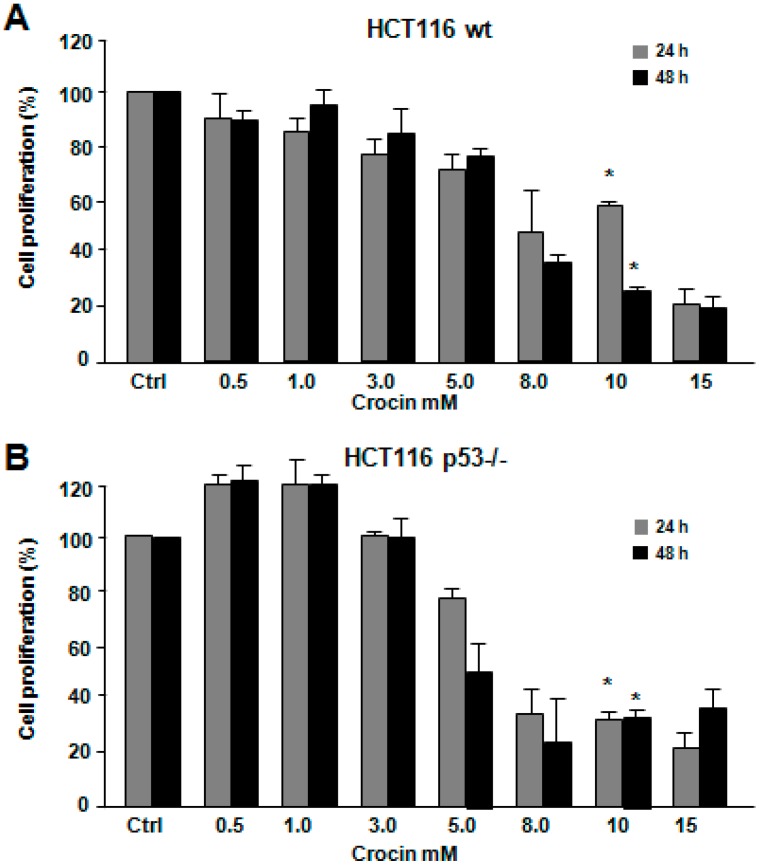

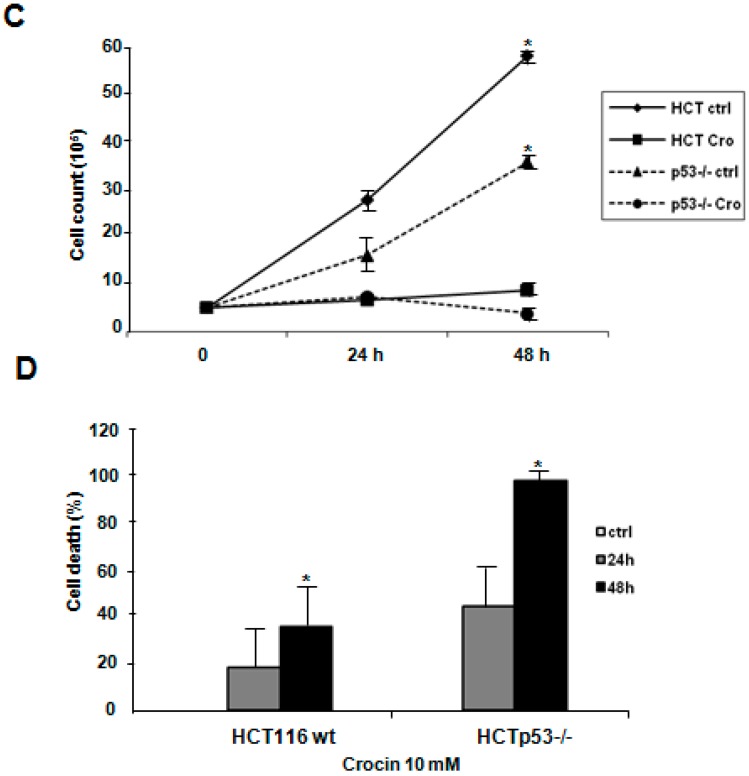
Effect of increasing concentrations of crocin on the growth of HCT116 wild-type (wt) and HCT116 p53−/− cells for 24 and 48 h. (**A**,**B**) Viability test assessed by 3-(4,5-dimethylthiazol-2-yl)-2,5-diphenyltetrazolium bromide (MTT) showing the HCT116 wild-type and HCT116 p53−/− cells untreated (ctrl) or treated with different concentrations of crocin (Cro; 0.5 to 15 mM) for 24 and 48 h. The MTT data shown are performed in quadruplicates; and (**C**) Cell numbers (dead and alive) and (**D**) cell death percentages were measured using the trypan blue staining after crocin treatment for 24 and 48 h (* *p* < 0.05).

The effect of crocin on HCT116 cell lines’ cell viability was examined by MTT analysis. Crocin reduced proliferation in a time- and dose-dependent manner. Figure 1A,B show a significant, but different cell proliferation inhibition (*p* < 0.05) of both HCT116 wild-type and HCT116 p53−/− cell lines at a concentration of 10 mM of crocin after 24 h, of 40% and 65%, respectively. However, both cell lines showed the same pattern of reduced cell proliferation (65%) after 48 h of crocin treatment. To justify this result, trypan blue staining was conducted for both cell lines treated with 10 mM crocin for 24 and 48 h. Indeed, crocin induced more cell death among the surviving 30% (Figure 1B) HCT116 p53−/− cells compared to HCT116 wild-type cells after 24 and 48 h (Figure 1C,D). This is in agreement with a previous study showing that crocin-mediated cell viability inhibition was affected by p53 status [24].

### 2.2. Different Method of Cell Cycle Arrest in HCT116 Cells after Crocin Treatment

**Figure 2:**
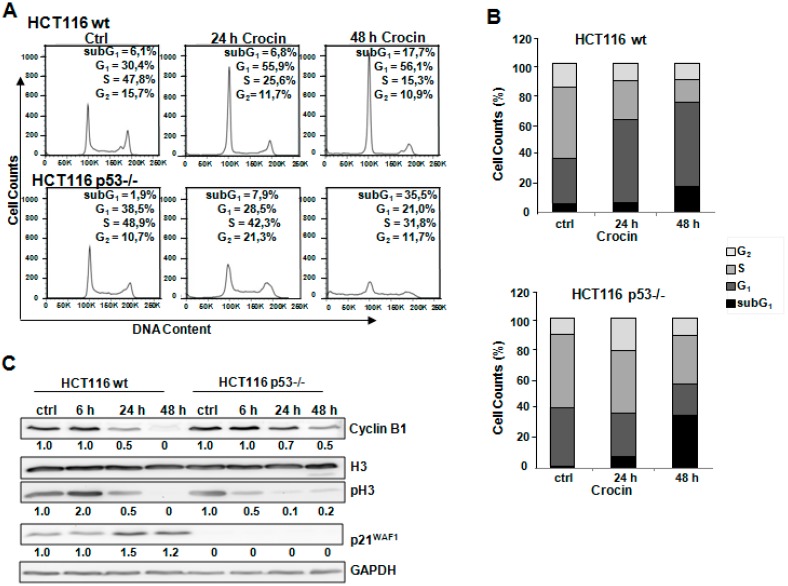
Cell cycle arrest after crocin administration. (**A**) Two cell types, HCT116 wild-type (wt) and HCT116 p53−/−, untreated (Ctrl) or treated with 10 mM crocin for 24 and 48 h, were harvested and DNA was stained with PI for flow cytometric analysis of DNA content with FACScan flow cytometry; (**B**) Quantitative analysis of percentage gated cells at sub-G_1_, G_1_, S, and G_2_ phases in the HCT116 wild-type and HCT116 p53−/− cells treated with 10 mM crocin for 24 and 48 h; and (**C**) Lysates prepared from two cell types, HCT116 wild-type and HCT116 p53−/−, untreated (Ctrl) or treated with 10 mM crocin for 6, 24, and 48 h were analyzed by anti-H3, anti-phospho Histone 3 (pH3), anti-Cyclin B1, anti-p21^WAF1^, and anti-GAPDH via Western blotting. GAPDH served as the internal control for equal loading. The ratios represent protein alterations compared to the control.

Flow cytometric analysis of cell cycle distribution revealed that there was an accumulation of HCT116 wild-type cells in the G_1_ phase of the cycle (55.9%, 56.1%) compared to the control (30.4%) after 24 and 48 h of crocin treatment, respectively (Figure 2A,B). In agreement with a previous study [25], there was an increase in p21WAF1 protein levels after crocin treatment in p53-expressing cells, suggesting its role in the observed G_1_ arrest (Figure 2C). This finding was further confirmed by the continuous decrease of the cell cycle regulators phospho-H3 and Cyclin B1 (Figure 2C). On the other hand, there was a different pattern of cell cycle distribution in the p53-deficient cells. Figure 2A,B show that crocin induced G_2_ arrest in HCT116 p53−/− after 24 h. Furthermore, 48 h treatment with crocin reinforced the cells to enter M phase and eventually undergo cell death (pre-G_1_ 35.5%) compared to the control (1.9%). The reduction of phospho-H3 would explain why cells stopped cycling after 24 h of crocin treatment (Figure 2C). Crocin treatment after 24 h induced G_2_ arrest, which was evaluated by reduction in the Cyclin B1 protein level (Figure 2C). At a later time point, there was an increase in phospho-H3 accompanied by regular levels of Cyclin B1 that cells might start recycling after 48 h of crocin treatment, which led to a delayed cell death.

### 2.3. Crocin Induces p53-Dependent Apoptosis in HCT116 Cells

**Figure 3:**
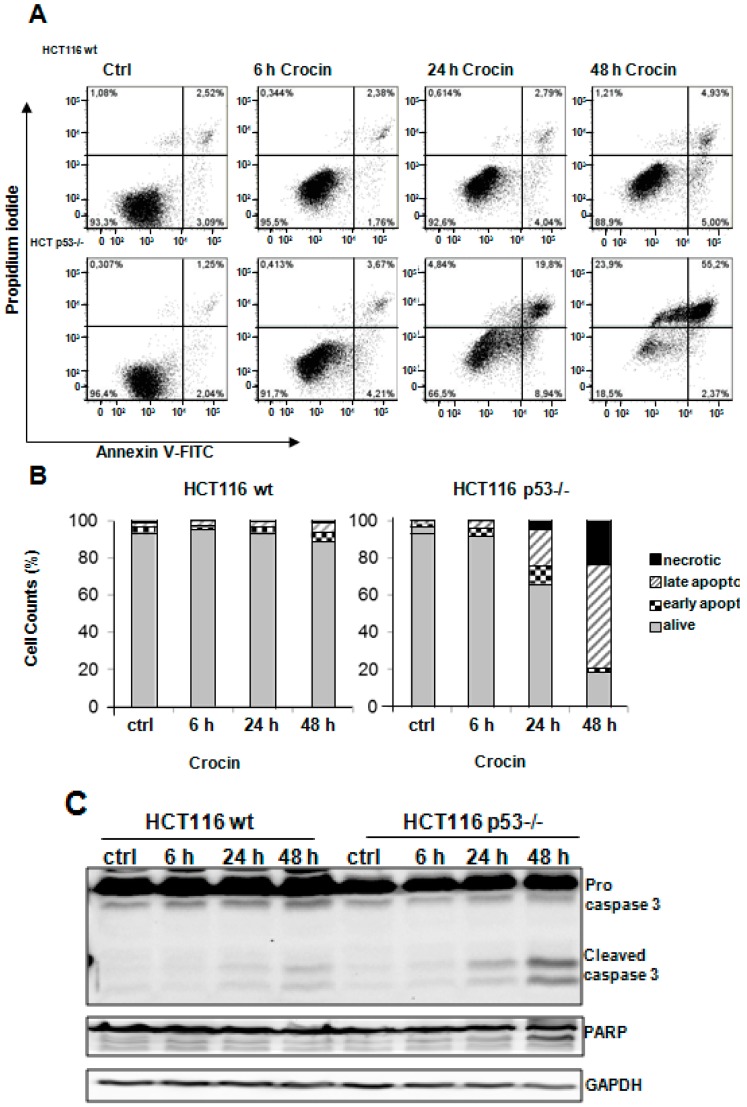

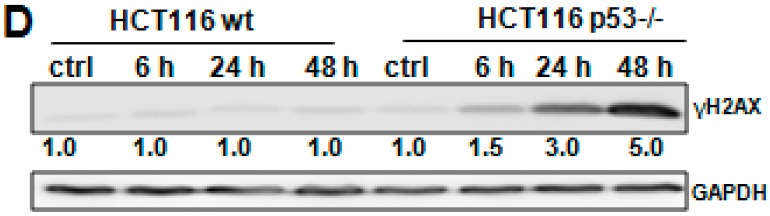
Apoptosis induction after crocin treatment. (**A**) Annexin-PI measurements of untreated cells (Ctrl) and two cell types, HCT116 wild-type (wt) and HCT116 p53−/−, treated with 10 mM crocin for 6, 24, and 48 h. The profile represents Annexin-V-FITC staining on the *x*-axis and PI on the *y*-axis; (**B**) Quantitative analysis of percentage gated for viable, necrotic, early apoptotic, and late apoptotic HCT116 wild-type (wt) and HCT116 p53−/− cells treated with 10 mM crocin for 6, 24, and 48 h; (**C**) Lysates prepared from two cell types, HCT116 wild-type (wt) and HCT116 p53−/−, untreated (Ctrl) or treated with 10 mM crocin for 6, 24, and 48 h, were analyzed by anti-caspase3, anti-PARP, and anti-GAPDH Western blotting. GAPDH served as the internal control for equal loading; and (**D**) Lysates prepared from two cell types, HCT116 wild-type (wt) and HCT116 p53−/−, untreated (Ctrl) or treated with 10 mM crocin for 6, 24, and 48 h were analyzed by anti-γH2AX, and anti-GAPDH Western blotting. GAPDH served as the internal control for equal loading. The ratios represent protein alterations compared to the control.

Next we aimed to analyze the possible pro-apoptotic effect of crocin on HCT116 cells by performing Annexin V staining. As shown in Figure 3A,B, there was a pronounced cell death induction in HCT116 p53−/− cells (63%) after 48 h of crocin treatment compared to HCT116 wild-type (11%). Analyzing the caspase 3 protein levels further proved that HCT116 p53−/− cells were more sensitive to crocin treatment. Figure 3C indicates a remarkable cleavage of caspase 3 in HCT116 p53−/− cells, which was associated with a cleavage increase in its downstream target, PARP, compared to the HCT116 wild-type after crocin treatment. Moreover, there was no indication of DNA damage, measured by the amount of γH2AX in the HCT116 wild-type cells after crocin treatment (Figure 3D). As expected, in p53-deficient cells, the observed cell death was accompanied by an increased DNA damage detected by a notable increase in γH2AX, which was significantly intensified after 6 h of crocin treatment in a time-dependent manner (Figure 3D).

### 2.4. Crocin Induces Aberrant and Inefficient Autophagy in HCT116 p53−/− Cells

**Figure 4:**
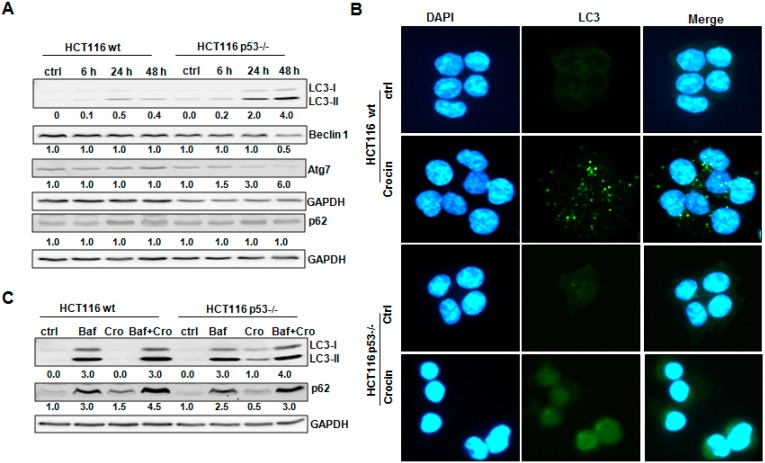
Crocin-induced autophagy in HCT116 cells. (**A**) Lysates prepared from two cell types, HCT116 wild-type (wt) and HCT116 p53−/−, untreated (Ctrl) or treated with 10 mM crocin for 6, 24, and 48 h, were analyzed by anti-LC3, anti-Beclin 1, anti-Atg7, anti-p62, and anti-GAPDH Western blotting. GAPDH served as the internal control for equal loading; (**B**) Fluorescence staining of LC3 and DAPI in two cell types, HCT116 wild-type (wt) and HCT116 p53−/−, control and treated with 10 mM crocin for 24 h; and (**C**) Lysates prepared from two cell types, HCT116 wild-type (wt) and HCT116 p53−/−, untreated (Ctrl) or treated with Bafilomycin A1 (Baf, 10 nM) for 1 h and/or further treated with 10 mM crocin (Cro) for 48 h were analyzed by anti-LC3, anti-p62, and anti-GAPDH Western blotting. GAPDH served as the internal control for equal loading. The ratios represent protein alterations compared to the control.

We have previously shown that p53 played a role in autophagy induction after saffron treatment [8]. Since crocin is a major active ingredient of saffron [26], we aimed to examine whether the p53 status could mediate autophagy induction in HCT116 cells, by analyzing autophagy-regulatory proteins LC3, Beclin 1, Atg7, and p62. As shown in Figure 4A, there was a conversion of LC3-I to LC3-II in HCT116 wild-type cells after crocin treatment, which was associated with unchanged protein levels of Beclin 1 and Atg7. Moreover, we did not detect any dramatic degradation of p62 (Figure 4A). Although autophagosome formation was confirmed by measuring LC3-II staining by immunofluorescence staining (Figure 4B), we suggest that there was only a slight autophagy induction after crocin treatment. In contrast to HCT116 wild-type cells, crocin induced a remarkable formation of LC3-II in HCT116 p53−/− cells after 24 and 48 h, which was combined with a decrease in the protein levels of Beclin 1and Atg7 later after 48 h, whereas there was no clear p62 degradation at any time point (Figure 4A). Surprisingly, although there was more LC3-II conversion, we did not observe any punctuation of LC3-II staining in HCT116 p53−/− after crocin treatment (Figure 4B), predicting a non-functional autophagosome formation.

In order to ascertain a possible autophagy function after crocin treatment, we assessed the autophagic flux. For this we treated cells with Bafilomycin A1 (Baf), an inhibitor of autophagosome and lysosome fusion [27]. It is known that accumulation of LC3-II might be a consequence of new formation of LC3 or hindering its degradation [28]. Indeed, HCT116 wild-type cells showed a crocin-induced increase of LC3-II levels, which was further strengthened after Baf treatment (Figure 4C). Furthermore, there was a remarkable increase of p62 after treating the cells with Baf and crocin compared to crocin exposure alone (Figure 4C), confirming an effective autophagic flux. In contrast, inhibiting fusion of lysosomes to autophagosomes in HCT116 p53−/− cells exposed to crocin by Baf treatment did not change the levels of LC3-II, suggesting defective autophagosome formation (Figure 4C). In addition, only a slight increase in p62 could be observed in cells treated with both Baf and crocin, which corresponds to defective autophagic flux (Figure 4C).

### 2.5. Crocin Modulation of HCT116 Cells’ Fate Was p53-Dependent

**Figure 5:**
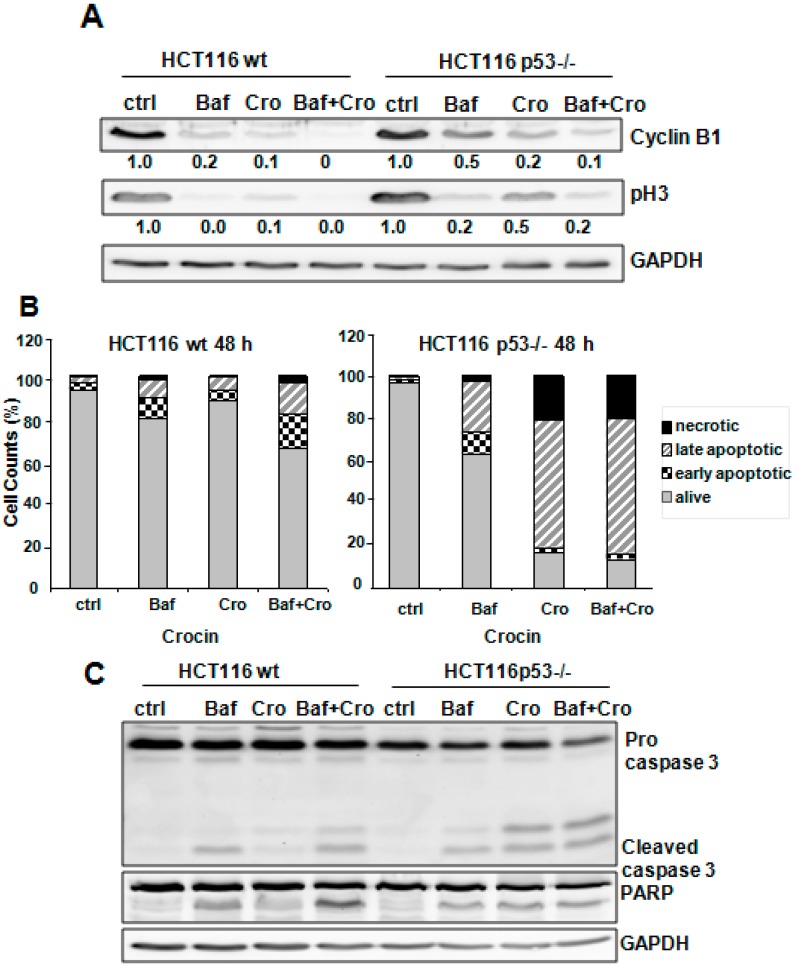

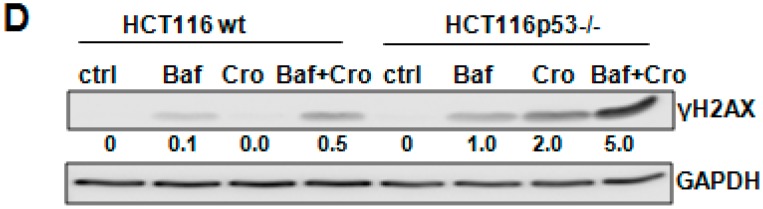
Crocin-induced autophagy in a p53-dependent manner. (**A**) Lysates of HCT116 wildtype and HCT116 p53−/−, untreated (Ctrl) or treated with Bafilomycin A1 (10 nM) for 1 h and/or further treated with 10 mM crocin for 48 h, were analyzed by anti-cyclin B1, anti-phospho Histone 3 (pH3) and anti-GAPDH via Western blotting. GAPDH served as internal control for equal loading; (**B**) Quantitative analysis of percentage gated viable, necrotic, early apoptotic, and late necrotic cells in the HCT116 wild-type (wt) and HCT116 p53−/− cells treated with Bafilomycin A1 (Baf, 10 nM) for 1 h and/or further treated with 10 mM crocin (Cro) for 48 h, measured by Annexin-PI staining; (**C**) Lysates prepared from two cell types, HCT116 wild-type (wt) and HCT116 p53−/−, untreated (Ctrl) or treated with 10 mM crocin for 24 h and/or further treated with bafilomycin (10 nM) for 48 h, were analyzed by anti-caspase3, anti-PARP, and anti-GAPDH Western blotting. GAPDH served as the internal control for equal loading; and (**D**) Lysates prepared from two cell types, HCT116 wild-type (wt) and HCT116 p53−/−, untreated (Ctrl) or treated with Bafilomycin A1 (10 nM) for 1 h and/or further treated with 10 mM crocin for 48 h, were analyzed by anti-γH2AX and anti-GAPDH in Western blotting. The ratios represent protein alterations compared to the control.

It is widely known that autophagy could play a role in either anti-apoptotic or pro-apoptotic pathways. Annexin V staining was analyzed to explore whether autophagy might be involved in the observed different patterns of induced apoptosis after Baf treatment and with or without crocin exposure. Figure 5A shows that Baf pretreatment enhanced the apoptosis induction in HCT116 wild-type cells, whereas there were only minor changes in the massive induced cell death in HCT116 p53−/− after crocin exposure. Moreover, inhibiting autophagosome-lysosome complex formation in HCT116 wild-type cells led to a pronounced cleavage of caspase 3 and PARP, which was associated with an increase in γH2AX protein levels after crocin exposure, suggesting an autophagy-driven pro-survival role of crocin when p53 is functional (Figure 5B). Conversely, Baf-exposed HCT116 p53−/− cells did not show a notable enhancement of apoptosis induction through an increased cleavage of caspase 3 or PARP after crocin treatment (Figure 5B). The increase in the DNA damage sensor γH2AX may suggest a pro-survival role of autophagy (Figure 5C). Thus, crocin induced apoptosis or autophagy, depending on the p53 status of the cells.

## 3. Discussion

In the present study, the crocin-induced decrease in cell proliferation was associated with induction of apoptosis in both tested p53 isogenic HCT116 cells. The crocin-induced cell death in HCT116 p53−/− cells was associated with DNA damage, as reflected by the upregulation of the double-stranded DNA breakage marker γH2AX. Crocin may therefore play an important role in sensitizing cancer cells to the effects of other chemotherapeutics. We have most recently reported similar effects for saffron [8].

Cell-cycle analysis revealed that crocin blocked the proliferation of HCT116 wild-type cells in the G_0_/G_1_ phase of the cell cycle and ultimately caused apoptosis-dependent cell death. Unlike its effect on HCT116 wild-type cells, crocin stopped the cell cycle progression of HCT116 p53−/− cells in the G_2_/M phase. Bajbouj *et al.* [8] have documented a similar G_2_ cell cycle arrest effect caused by saffron crude extract on HCT116 cells. Similarly, the fungal-based cordycepin was shown to induce G_2_/M cell-cycle arrest in an oral squamous cancer cell line [23,29]. Further examination of molecular markers associated with G_1_ arrest showed remarkable changes, including increased p21 level and reduced Cyclin B1 and phospho-H3 levels (Figure 2), in crocin-treated HCT116 wild-type cells. Western blot analyses suggested that crocin markedly induced differential cell-cycle arrest in both tested cell lines. It has also been reported that G_2_/M phase cell cycle arrest can be mediated by the p21 cell cycle inhibitor [30,31], as well as independently of p21 activation [32,33]. The latter seems to be the case with the anti-proliferative activity of crocin in the present study, as the drug failed to increase expression of p21 in HCT116 p53−/− cells. Crocin has effectively inhibited tumor cell growth concomitant with induction of cell death, particularly in HCT116 p53−/− cells (Figure 3). The cytotoxicity of many anticancer agents is mediated by autophagy activation, which is associated with cell cycle arrest [34,35,36]. Autophagy has also been reported as a protective mechanism to counteract the cellular stress induced by chemotherapy [37]. Autophagy has also been a contributing factor in cell death when energy is required. Alternatively, in developmental systems autophagy has been shown to indirectly facilitate cell death [38]. Thus, the overall situation is complex, and the potential benefit of inhibition of autophagy seems likely to vary between individual tumor types and even within the same tumor over time [37]. Two hypotheses have been put forward to address such irony. One suggests different roles of autophagy depending on different stages of tumor development. Thus, autophagy slows down tumor formation at an early stage, but favors tumor cell survival and invasion as soon as cancer has formed. The second hypothesis confers tissue specificity as the main regulator of autophagy during carcinogenesis [39].

The present study shows that p53 status mediates autophagy induction in HCT116 cells via modulating different autophagy-regulatory proteins including LC3, Beclin 1, Atg7, and p62. To obtain better insight into the mechanism of crocin-induced autophagy, we analyzed the effects of crocin on LC3-II protein, the lipidated form of mammalian microtubule-associated protein 1 light chain LC3-I. LC3-II is expressed during autophagosome formation and its accumulation is a hallmark of autophagy [36,40]. Crocin treatment of HCT116 wild-type cells resulted in a notable upregulation of the LC3-II protein, which was associated with unchanged protein levels of Beclin 1 and Atg7. In contrast, the crocin-induced formation of LC3-II in HCT116 p53−/− cells was combined with a decrease in the protein levels of Beclin 1 and Atg7 (Figure 4A). Crocin-induced autophagosome formation was also demonstrated by measuring punctuation of LC3-II immunofluorescence staining. Vesicular organelles (autophagosomes) were formed in crocin-treated HCT116 wild-type cells, as indicated by LC3 staining, but were not detected in HCT116 p53−/− after crocin treatment (Figure 4B), predicting a nonfunctional autophagosome formation. LC-3II accumulates when the autophagosome-lysosome fusion process or the downstream lysososomal degradation step is impaired, as LC3-II itself is subject to degradation due to its localization to autophagosomes [38,41]. This possibility was excluded by using the autophagy inhibitor Bafilomycin A1, an inhibitor of autophagosome and lysosome fusion [27] that was shown to enhance crocin-induced LC3-II accumulation in HCT116 wild-type cells to a greater extent than either agent alone, indicating that the accumulation of LC3-II was from intact autophagic flux and consistent with autophagy induction [42]. Furthermore, there was a remarkable increase in p62 after treating the cells with Baf and crocin compared to crocin exposure alone (Figure 4C). p62 is localized at the autophagic compartments and is normally degraded through autophagy; the crocin-induced increase of p62 suggests a crocin-mediated impairment of the autophagic degradation process. On the contrary, inhibiting fusion of lysosomes to autophagosomes in HCT116 p53−/− cells exposed to crocin did not change the levels of LC3-II (Figure 4C). That corresponded well to the low Beclin 1 and Atg7 levels, which would normally have led to an effective double membrane formation and hence a functional autophagosome. The slight increase in p62 in cells treated with both Baf and crocin might be due to partial Baf-induced impairment of autophagosome degradation (Figure 4C).

Although the interplay between apoptosis and autophagy remains ambiguous, the cellular context is crucial. In fact, three hypotheses have been introduced to describe the rapport between autophagy and apoptosis. First, autophagy is critical for apoptosis to occur and would eventually lead to cell death. Second, autophagy antagonizes apoptosis, permitting cells to survive. Third, apoptosis and autophagy coexist and both determine the final fate of cells [43]. The addition of the autophagy inhibitor Bafilomycin A1 differentially affected crocin-induced apoptosis (Figure 5). Whereas Baf alone had little effect on apoptotic cell death, pretreatment of HCT116 wild-type cells with Baf significantly increased apoptosis, suggesting a cytoprotective (pro-survival) role of autophagy in HCT116 wild-type cells that perhaps allows cells to escape from apoptosis. In fact, the cellular context has been shown to fine-tune autophagy as a protective mechanism for malignant cells [34] or as a promoter of antineoplastic responses [44]. In contrast, several recent investigations showed that inhibition of the autophagy intensified apoptosis, indicating that autophagy may be a protective response against anti-cancer agents that contributes to tumor progression [45,46]. In this aspect, the autophagic pathway is a novel therapeutic target for cancer treatment [18]. Autophagy inhibition also enhances the anticancer effect of arsenic trioxide and hyperthermia [47], sulforaphane [48], and p53 or alkylating drugs [36].

In p53 wild-type cells, the Baf-mediated augmentation of crocin-induced apoptosis was associated with DNA damage, cleavage of PARP, and activation of caspase 3. On the other hand, Baf-exposed HCT116 p53−/− cells showed a subtle induction of apoptosis after crocin treatment, indicating that crocin induced classical programmed cell death, which was autophagy-independent. Inhibition of p53 was shown to enhance autophagy and improve the survival of p53-defective cells by sustaining high levels of ATP. p53-induced autophagy may therefore contribute to cell cycle arrest and DNA repair by selective degradation of damaged molecules and organelles in order to provide an energy source for the DNA damage repair. However, when the DNA damage is beyond repair, autophagy may speed up cell death as a result of p53 activation.

## 4. Experimental Section

### 4.1. In Vitro Analysis

HCT116 colon cancer cells; both p53 wild-type and p53−/− were purchased from American Type Culture Collection (Manassas, VA, USA). Colon cancer cells were kept in a humidified CO_2_ incubator at 5% CO_2_ and 95% room air, and were grown in appropriate growth media. Used media were supplemented with 10% FBS, 100 U/mL penicillin, and 100 μg/mL streptomycin. Cells were periodically sub-cultured with 1:250 (trypsin/EDTA; PAA Laboratory, Colbe, Germany). Different crocin concentrations were tested in both cell types at different time points.

### 4.2. MTT Cell Proliferation Assay

MTT (3-(4,5-dimethylthiazol-2-yl)-2,5-diphenyltetrazolium bromide; Sigma-Aldrich, Steinheim, Germany) was used as a colorimetric assay to assess cell viability. It was utilized in examined HCT116 cell lines to evaluate crocin’s effects on cell proliferation. Ten thousand cells were grown in 0.2 mL growth medium in 96-well microtiter plates. Cell were allowed to attach overnight and were then treated with increasing concentrations of crocin starting at 0.5, 1, 3, 5, 8, 10, and 15 mM for 24 and 48 h. All experiments were conducted thrice and were repeated independently at least three times. Judging by how much purple formazan (MTT final product) was made, growth of living cells was quantified. MTT was then mixed with colon cancer cells at 37 °C for 2 h in a humidified CO_2_ incubator at 5% CO_2_. MTT formazan product was dissolved in DMSO and absorbance was then measured at 570 nm in a microplate reader (SpectraFluor, Tecan, Männedorf, Switzerland).

### 4.3. Crystal Violet Assay

The cytotoxicity effect of crocin on HCT116 cells was evaluated using crystal violet assay. Cells (7.5 × 10^3^) were seeded in 96-well microtiter plates and were grown in 0.2 mL of the appropriate growth medium. After an overnight attachment period, cells were treated with crocin, as detailed above, in an MTT assay. Phosphate-buffered saline (PBS) was used to wash cells that were then incubated with DNA stain at 50 μL (staining solution: 0.5% crystal violet, 20% methanol) and placed on a shaker at Room Temperature (RT). The plate was double washed with distilled water and was then allowed to dry. After 15 min incubation on a shaker, methanol (200 microliters) was used to dissolve the uptaken crystal violet. The dye taken up by the treated cells was quantified by reading the absorbance at 570 nm in a microplate reader. All experiments were carried out and repeated independently at least three times.

### 4.4. Fluorescence Activated Cell Sorting (FACS) Analysis of DNA Contents

Cells (1.2 × 10^6^ cells per well of a 6-well plate) were allowed to grow a day before crocin treatment. Cells were then trypsinized, harvested, washed twice in PBS, suspended in 0.5 mL ice-cold PBS, and fixed overnight with 4 mL of ice-cold 70% ethanol. An extraction buffer composed of nine parts 50 mM Na_2_HPO_4_, one part 25 mM citric acid, and 0.1% Triton X-100 at a pH of 7.8 was used to extract low molecular weight DNA fragments after 10 min incubation. Cells were spun down, resuspended, and incubated in the dark at RT in 0.4 mL staining buffer (pH 6.8; 10 mM PIPES, 100 mM NaCl, 2 mM MgCl_2_, 0.1% Triton X-100) with the addition of 250 μg RNase and propidium iodide (50 μg/mL final concentration). The DNA contents and distribution of different cell cycle phases were analyzed using a flow cytometer FACS CantoII (Becton-Dickinson, San Diego, CA, USA). Sub G_1_ cell population in the FACS histograms were considered apoptotic cells. Cell cycle distribution and the differential percentages of cell populations in the different phases (G_1_, S, and G_2_/M) of the cell cycle were assessed using the cell cycle platform of the FlowJo software and the Watson pragmatic model (Tree Star, San Carlos, CA, USA).

### 4.5. Apoptotic Assessment Using Annexin V-PI Staining

An Annexin V-FLUOS kit from Roche Diagnostics (Indianapolis, IN, USA) was utilized to assess apoptosis. Cells at a density of 1 × 10^6^ treated for 6, 24, and 48 h with crocin were washed twice in PBS. Cells were stained for 15 min at RT with 0.1 mL annexin V staining solution, made up of 20 μL FITC-conjugated annexin V reagent (20 μg/mL), 20 μL isotonic propidium iodide (50 μg/mL), and 1000 μL of 1 M/L HEPES buffer. Using flow cytometer FACS CantoII (Becton-Dickinson) at 488 nm excitation, a 530/30 nm band pass filter for fluorescein detection, and a long pass filter 2P670 nm, cells were analyzed for propidium iodide detection after electronic compensation. As cells with positive annexin V staining may indicate apoptosis and necrosis, cells positive for propidium iodide were assessed to show late apoptotic cells and necrotic cells. Cells that were annexin V-positive and propidium iodide-negative were, however, counted as early apoptotic cells.

### 4.6. Western Blotting

Whole cell lysates were prepared from both examined types of colon cancer cells. Bio-Rad DC Protein Assay (BioRad Laboratories, Hercules, CA, USA) was used to determine the protein concentration of lysates. Thirty-μg proteins were loaded onto 12% SDS polyacrylamide gel electrophoresis (PAGE). Gels were then transferred onto nitrocellulose membranes. Different primary antibodies was utilized in this study including antibodies against H3 (Active Motif, Carlsbad, CA, USA), LC3 (Nanotools, Teningen, Germany), Cyclin B1 (Santa Cruz Biotechnology, Santa Cruz, CA, USA), phospho-H2AX (Millipore, Billerica, MA, USA), caspase 3, phospho-H3 (pH3), PARP, p21^WAF1^, Atg7, Beclin 1, and p62 (all from Cell Signaling Technology Inc., Beverly, MA, USA). Secondary antibodies (anti-mouse or anti-rabbit IgG peroxidase conjugated; Pierce, Rockford, IL, USA) were also utilized. Bound antibodies were detected by incubating the blots in West Pico chemiluminescent substrate (Pierce). Levels of immunoreactivity were assessed as peaks intensity using an image capture and analysis system (GeneGnome, Syngene, UK). Anti-GAPDH antibody was used to control protein quality and equal loading.

### 4.7. Immunofluorescence Labeling

Cells (3 × 10^4^) per well seeded on coverslips control and treated with 10 mM crocin for 24 h were fixed in 3% paraformaldehyde for 15 min on ice. Cells were permeabilized in 1 mL 0.2% Triton X-100 for 5 min at room temperature. The cells were then washed at room temperature three times with PBS. Afterwards, the cells were blocked with 1 mL of 3% normal goat serum dissolved in 0.1% Triton X-100 /PBSX-100/PBS for 20 min at RT. The subsequent reaction was carried out first by staining the cells with anti-LC3 at dilution of 1:1000 overnight at 4 °C, followed by a 1:500 dilution of Alexa-488 labeled goat-anti-mouse-IgG secondary antibody (Vector Labs, Burlingame, CA, USA) for 10 min at room temperature. The slides were counterstained and mounted with 4'-6-Diamidino-2-phenylindole 40-6-Diamidino-2-phenylindole DAPI as a nuclear marker + mounting medium (Vector Labs) and were examined under a fluorescence microscope equipped with the appropriate filters.

### 4.8. Statistical Analysis

Data were analyzed using a Student’s *t*-test to calculate the significance values; a probability value *p* < 0.05 was considered statistically significant.

## 5. Conclusions

In conclusion, the golden spice’s main bioactive ingredient, crocin, is shown here to differentially inhibit proliferation of both HCT116 wild-type and HCT116 p53−/− cell lines. HCT116 wild-type cells were arrested in G_1_ after crocin treatment, whereas it caused a mild G_2_ arrest in HCT p53−/−. Thus, at least up to 48 h, a cytostatic rather than cytotoxic effect of crocin seems to mediate the survival of HCT wild-type cells. Pre-treatment with Baf, an inhibitor of autophagosome and lysosome fusion, enhanced the induction of apoptosis in HCT116 wild-type cells but not in HCT116 p53−/− cells, indicating that crocin induced an autophagy-independent classical programmed cell death.
